# Multi-crystal native-SAD phasing at 5 keV with a helium environment

**DOI:** 10.1107/S205225252200971X

**Published:** 2022-10-21

**Authors:** Akira Karasawa, Babak Andi, Martin R. Fuchs, Wuxian Shi, Sean McSweeney, Wayne A. Hendrickson, Qun Liu

**Affiliations:** a Center on Membrane Protein Production and Analysis, New York Structural Biology Center, New York, NY 10027, USA; b Photon Sciences, NSLS-II, Brookhaven National Laboratory, Upton, NY 11973, USA; c Department of Biochemistry and Molecular Biophysics, Columbia University, New York, NY 10032, USA; d Department of Physiology and Cellular Biophysics, Columbia University, New York, NY 10032, USA; e Biology Department, Brookhaven National Laboratory, Upton, NY 11973, USA; Chinese Academy of Sciences, China

**Keywords:** anomalous diffraction, native-SAD, low energy, helium paths, membrane proteins, multiple crystals

## Abstract

*De novo* structure determination from single-wavelength anomalous diffraction using native sulfur or phospho­rus in biomolecules (native-SAD) is an appealing method to mitigate the labor-intensive production of heavy-atom derivatives and seleno­methio­nyl substitutions. The native-SAD method is particularly attractive for membrane proteins, which are difficult to produce and often recalcitrant to grow into decent-sized crystals. Native-SAD uses lower-energy X-rays to enhance anomalous signals from sulfur or phospho­rus. However, at lower energies, the scattering and absorption of air contribute to the background noise, reduce the signals and are thus adverse to native-SAD phasing. We have previously demonstrated native-SAD phasing at an energy of 5 keV in air at the NSLS-II FMX beamline. Here, the use of a helium path developed to reduce both the noise from background scattering and the air absorption of the diffracted X-ray beam are described. The helium path was used for collection of anomalous diffraction data at 5 keV for two proteins: thaumatin and the membrane protein TehA. Although anomalous signals from each individual crystal are very weak, robust anomalous signals are obtained from data assembled from micrometre-sized crystals. The thaumatin structure was determined from 15 microcrystals and the TehA structure from 18 microcrystals. These results demonstrate the usefulness of a helium environment in support of native-SAD phasing at 5 keV.

## Abstract 2

Low-energy native-SAD phasing using a helium path could be used more routinely for solving challenging membrane protein structures.

## 1. Introduction

Macromolecular X-ray crystallography is the mainstay technology for structure determination, accounting for nearly 90% of all PDB deposits as of 1 June 2022. (https://www.rcsb.org/stats/summary). However, each structure determination must solve the phase problem in order to transform X-ray diffraction intensities into molecular images. Molecular replacement (MR) is the dominant method to solve the phase problem [for approximately 80% of all PDB deposits (Jin *et al.*, 2020 ▸)]. However, MR cannot be used for macromolecules without a structurally similar model. This situation is exacerbated for membrane proteins: about 30% of the human genome contains codes for membrane proteins (Wallin & Heijne, 1998 ▸), however only 3.7% of deposited structures in the PDB are membrane proteins (http://pdbtm.enzim.hu/?_=/statistics/growth) and many of them are from prokaryotes and have low sequence identity to their structurally uncharacterized eukaryotic homologs. Indeed, half of all the unique membrane protein structures were determined by *de novo* structure determination, rather than MR (Huang *et al.*, 2018 ▸). Even when there is a similar structure model or a predicted model by *AlphaFold* (Jumper *et al.*, 2021 ▸) or *RoseTTAFold* (Baek *et al.*, 2021 ▸), conformational changes and model bias may compromise the structure determination and refinement (Kleywegt, 2000 ▸). Therefore, *de novo* structure determination remains a requirement for the structure determination of many proteins.

Single-wavelength anomalous diffraction (SAD) is the most commonly used method for *de novo* structure determination (Wang, 1985 ▸; Chen *et al.*, 1991 ▸; Cowtan & Main, 1993 ▸; Hendrickson, 2014 ▸). SAD phasing depends on the anomalous scattering factor *f*″ (Liu & Hendrickson, 2017 ▸) and it has been mostly performed on heavy-atom (*Z* > 20, such as Hg, Au, Ir) derivatives of protein crystals by heavy-atom soaking, co-crystallization or seleno­methio­nine (SeMet) substitution (Pike *et al.*, 2016 ▸). However, screening heavy-atom derivatives is time-consuming, and very often crystals may be resistant to derivatization, especially membrane proteins because of poor yield or low efficiency of SeMet incorporation (Pike *et al.*, 2016 ▸).

The alternative of *de novo* structure determination using intrinsic anomalous scatterers, such as sulfur in protein and phospho­rus in nucleic acids, is attractive because only native macromolecule crystals are needed, thus saving the complication of derivatization or SeMet substitution. This is called native-SAD (Liu *et al.*, 2012 ▸; Rose *et al.*, 2015 ▸). However, there are challenges associated with native-SAD phasing; only a small number of PDB deposits were determined by this method (Rose *et al.*, 2015 ▸; Weinert *et al.*, 2017 ▸). One challenge is that the sulfur *K*-edge is at 2.47 keV (Liu & Hendrickson, 2017 ▸), which is unreachable from the X-ray energies available at most synchrotron beamlines for biological crystallography. The second challenge at 2.47 keV is parallax effects from the use of flat detectors (Rose *et al.*, 2015 ▸). The third challenge is the absorption and scattering from air, which contribute to background, absorb faint signals and thus decrease signal over noise. When the energy of the X-rays is reduced towards the *K*-edge of sulfur or phospho­rus, *f*″ increases (Liu *et al.*, 2013 ▸). As a compromise, it is advantageous to carry out lower-energy native-SAD phasing at a higher energy. However, the benefits of lowered X-ray energy are offset by X-ray absorption and background scattering from air (Liu *et al.*, 2013 ▸; Wagner *et al.*, 2016 ▸). The most successful native-SAD phasing has been performed at an X-ray energy of around 6–7 keV (Mueller-Dieckmann *et al.*, 2005 ▸; Weinert *et al.*, 2015 ▸). To minimize these hurdles at energies below 6 keV, smaller sized crystals (∼20 µm or less) and an in-vacuum beamline (Aurelius *et al.*, 2017 ▸) or helium paths (Basu, Olieric *et al.*, 2019 ▸) can be used.

In addition to the X-ray energy, due to sample absorption the measurable anomalous signals from sulfur depend on the sample size (Liu *et al.*, 2012 ▸; Liebschner *et al.*, 2016 ▸, Basu, Olieric *et al.*, 2019 ▸). For large crystals (bigger than 100 µm in size), our calculations showed it to be advantageous to collect data at 6–7 keV in order to mitigate the sample absorption problem (Liu *et al.*, 2013 ▸). For small crystals (<50 µm), it might be better to use sub-6 keV X-rays to exploit enhanced transmitted anomalous signals. Indeed, Liebschner *et al.* (2016 ▸) showed the advantage of 4.6 keV over 6.5 keV for native-SAD phasing of ferredoxin reductase and lysozyme using crystals less than 100 µm in size. Thus, it appears attractive to use sub-6 keV X-rays for native-SAD phasing for a difficult structure determination as is typically the case for membrane proteins.

Here, we describe the use of a helium path developed at the National Synchrotron Light Source II (NSLS-II) FMX beamline (Schneider *et al.*, 2021 ▸) to improve the diffraction data signal-to-noise ratio at 5 keV. We also describe the procedure of successful native-SAD phasing for a soluble protein thaumatin and a challenging membrane protein TehA from fewer than 20 microcrystals.

## 2. Methods

### 2.1. Implementation of a helium path

**Figure 1:**
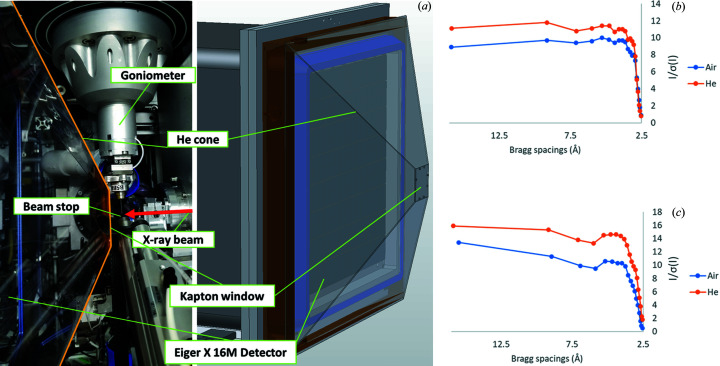
A helium environment for low-energy X-ray experiments. (*a*) The helium path setup at the FMX experimental station. The X-ray beam passes through the nitro­gen cooling gas stream after exiting the beam collimator tube 5 mm upstream of the crystal into the beamstop 5 mm downstream of the crystal. The scattered radiation passes through a Kapton window with a thickness of 7.62 µm at a distance of 12 mm downstream of the sample position. The detector surface of the EIGER X 16M detector is at 137 mm downstream of the crystal position. (*b*) and (*c*) Plot of 〈*I*/σ(*I*)〉 against *d*_min_. Highest signal-to-noise among single-crystal dataset of (*b*) thaumatin or (*c*) TehA included 1620 frames.

The FMX beamline is equipped with a DECTRIS EIGER X 16M detector, which has a detector shield to prevent accidental damage. To fit the detector size and to reduce air scattering and absorption for our experiments at 5 keV, we developed a pyramidal plastic helium path as shown in Fig. 1 ▸(*a*). The helium path is mounted on a frame surrounding the detector housing. It features an inlet on top and an outlet at the bottom of the chamber for helium flow, an oxygen sensor to verify fill levels, and one thin X-ray transparent Kapton window (7.62 µm thickness) on the upstream sample end. The distance between sample and detector is fixed at 137 mm, corresponding to a diffraction limit (*d*_min_) of 2.9 Å at the detector edge, and 2.5 Å in the detector corner for the 5 keV experiments. The beamline tungsten beamstop is 5 mm thick and is placed at a distance of 5 mm after the sample and 2 mm before the Kapton window (45 × 45 mm). So, the distance from the sample to the window is 12 mm in the beam direction.

### 2.2. Sample preparation

C-terminal His-tagged TehA was expressed in *E. coli* BL21(DE3) pLysS (Thermo Fisher Scientific) and purified by IMAC and size-exclusion chromatography as described by Chen *et al.* (2010 ▸). Purified TehA in 10 m*M* Tris pH 8.0, 200 m*M* NaCl, 1 m*M* EDTA, 0.5 m*M* TCEP and 50 m*M* octyl β-d-glucopyran­oside (β-OG), after removal of His-tag by TEV protease, was concentrated to 15 mg ml^−1^ with a Vivaspin 100 kDa cutoff (SARTORIUS) centrifugal concentrator. Concentrated TehA was supplemented with 10 m*M* spermidine then mixed with 0.1 *M* HEPES pH 7.8, 1 m*M* ZnSO_4_, 27–29% PEG 400 in a ratio of 1:1 (*v*/*v*) for crystallization by the sitting-drop method at 4°C. Microcrystals of about 20 × 20 × 20 µm grew overnight and were harvested by centrifugation at 500*g* for 5 min followed by removal of solution and resuspension with stabilization buffer consisting of 0.1 *M* HEPES pH 7.8, 1 m*M* ZnSO_4_, 27% PEG 400 and 50 m*M* β-OG in a cold room. Microcrystals were mounted in MiTeGen loops and cryo-cooled in liquid nitro­gen for diffraction data collection.

Thaumatin crystals were prepared as described previously (Guo *et al.*, 2018 ▸). Crystals with sizes of about 20 × 20 × 20 µm were mounted in MiTeGen loops and cryo-cooled in liquid nitro­gen for diffraction data collection.

### 2.3. Diffraction data collection

Microdiffraction data were collected at the FMX beamline at NSLS-II (Schneider *et al.*, 2021 ▸). We tuned the X-ray energy to 5 keV with a beam size of 5 × 6 µm (*V* × *H*). The measured beam flux was about 6 × 10^10^ photons s^−1^. Prior to data collection, the path was filled with helium gas as measured by the decreased level of oxygen using an oxygen sensor with a small, continuous flow after filling. Diffraction data were collected for each thaumatin or TehA crystal in rotation steps of 0.2° and an exposure time of 0.02 s per frame. At a sample-to-detector distance of 137 mm, the corresponding Bragg spacing was about 2.9 Å at the detector edge. The estimated X-ray transmission was 52% in the detector center and 36% at the detector edge. With the helium path filled, we collected 17 datasets for thaumatin and 23 datasets for TehA. For the purpose of comparing the impact of the helium path, we randomly selected 9 thaumatin crystals and 8 TehA crystals for the same data collection, but with air in the path.

### 2.4. Data reduction and assembly

Single-crystal datasets (total 1800 frames for each crystal and 0.2° oscillation per frame) were indexed and integrated independently as 10 accumulated wedges per crystal (1–180, 1–360, …, 1–1800) using *PyMDA* (Takemaru *et al.*, 2020 ▸) which integrates the software *DIALS*, *POINTLESS* and *AIMLESS* (Waterman *et al.*, 2016 ▸; Winter *et al.*, 2018 ▸; Evans, 2011 ▸; Evans & Murshudov, 2013 ▸). All wedges were scaled and the best wedges (CC_1/2_ value above 0.9 at 4 Å) were chosen for each crystal and then merged to 2.6 Å in *PyMDA* using its assembly module. To remove outlier crystals, crystal rejection was performed one-by-one in *PyMDA* from the assembled dataset for thaumatin (17 crystals) and TehA (23 crystals). The assembled datasets after each crystal rejection were further subjected to a different extent of frame rejection using *PyMDA*. The extent of frame rejection is defined using frame_cutoff = [min(SmRmerge) × (1 + decay)], where min(SmRmerge) is the lowest SmRmerge, reported by *AIMLESS*, within a single-crystal dataset; and decay is a rejection ratio of ∞ (no rejection), 500, 200, 150, 100 and 50%. Frames with SmRmerge larger than frame_cutoff were excluded from assembly in *AIMLESS*. For example, 200% indicates that frames with an SmRmerge of 200% more than min(SmRmerge) are rejected from scaling and merging (Guo *et al.*, 2019 ▸; Takemaru *et al.*, 2020 ▸). Fig. S3 of the supporting information summarizes the overall data reduction and assembly workflow.

### 2.5. Structure determination and anomalous scatterer calculations

The substructures of the anomalous scatterers were determined with *SHELXD* (Sheldrick, 2010 ▸) in the *CCP4I2* package (Potterton *et al.*, 2018 ▸). In total, 5000 trials were performed to search for anomalous scatterers in thaumatin and TehA data with *E*_min_ cutoffs between 1.3 and 1.7 and resolution cutoffs between 3.0 and 5.0 Å. The substructure which showed the highest *SHELXD* CC_all/weak_ values was used to calculate the initial SAD phases in *PHASER* (Read & McCoy, 2011 ▸), followed by density modification with *PARROT* (Cowtan, 2010 ▸). Model building was performed using *BUCCANEER* (Cowtan, 2006 ▸) for initial automatic model building, followed by iterative model building and refinement using *COOT* (Emsley & Cowtan, 2004 ▸) and *REFMAC5* (Murshudov *et al.*, 2011 ▸), respectively. Bijvoet-difference Fourier maps and peak heights were calculated using *ANODE* (Thorn & Sheldrick, 2011 ▸). The *f*″ values for anomalous scatterers were obtained through the *f*″ refinement (Liu *et al.*, 2013 ▸) using *phenix.refine* (Afonine *et al.*, 2012 ▸). We changed the occupancy of non-sulfur anomalous scatterers from 1 to 0.1 in 0.1 intervals and refined individual *B*-factor and *f*″ values. The refined *f*″ value of an anomalous scatterer is selected for an occupancy that has a *B*-factor value close to its neighboring atom(s). The mapCC between refined TehA or thaumatin and SAD-phased maps after density modification was calculated by model-map correlation in *Phenix* (Adams *et al.*, 2004 ▸).

## 3. Results

### 3.1. Diffraction data with a helium path at 5 keV

We developed a helium path for native-SAD phasing [Fig. 1 ▸(*a*)]. With a helium environment, we expected that the signal-to-noise ratio would be higher under helium than air. To examine the benefit in terms of signal-to-noise using this setup, we collected diffraction images from thaumatin and TehA crystals of the same size under helium and air environments. CC_1/2_ values under air and helium were very similar for each crystal [Figs. S1 ▸(*a*) and S1(*b*)], indicating that these datasets were valid for comparison. We found that the signal-to-noise ratio [〈*I*/σ(*I*)〉] under helium was higher than under air against any resolution in both cases [Figs. 1 ▸(*b*) and 1(*c*)]. Because we scaled the helium data with the air data as a reference, the increased 〈*I*/σ(*I*)〉 can be attributed to reduced X-ray absorption and decreased background noise, thus confirming that our helium setup reduced background scattering at 5 keV.

### 3.2. Native-SAD phasing of thaumatin

We first tested native-SAD phasing of thaumatin, which has been accomplished previously (Kim *et al.*, 2007 ▸; Nass *et al.*, 2016 ▸, 2020 ▸, 2021 ▸; Wagner *et al.*, 2016 ▸; Aurelius *et al.*, 2017 ▸; Leonarski *et al.*, 2018 ▸). Thaumatin consists of 207 residues, including 1 me­thio­nine and 16 cysteines forming 8 di­sulfide bridges. The calculated Bijvoet difference ratio (Hendrickson & Teeter, 1981 ▸) is 2.8% at 5 keV. We collected a total of 17 datasets with the helium path, each from a single crystal. Individually, none of them showed significant anomalous signals. To enhance anomalous signals from sulfur we performed crystal assembly and rejection using *PyMDA* (Takemaru *et al.*, 2020 ▸).

We chose a CC_1/2_ cutoff of 0.9 at 4 Å for each crystal to exclude radiation-damaged frames. We then analyzed unit-cell variations for all selected datasets. The thaumatin crystals showed similar unit-cell dimensions [Fig. S2(*a*)] indicating that all crystals are compatible for assembly. Therefore, we merged the 17 single-crystal datasets to enhance the anomalous signals.

**Figure 2:**
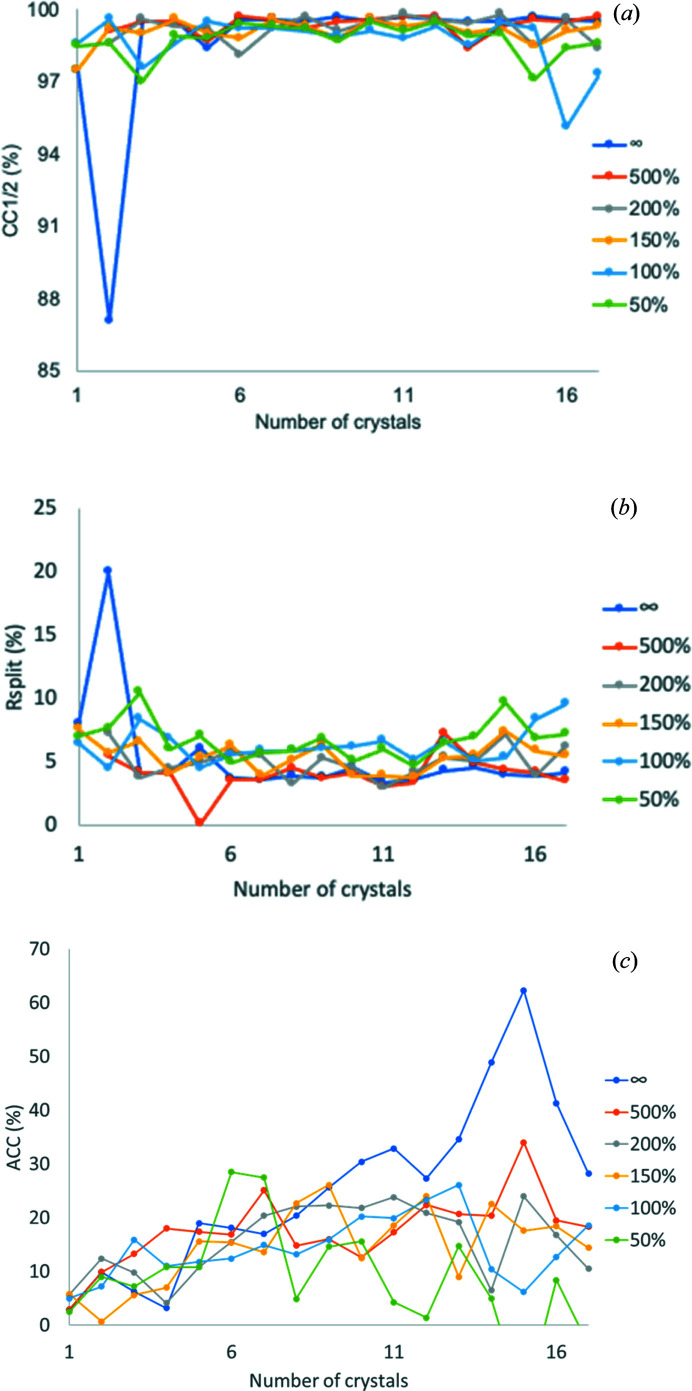
Data analysis of assembled datasets of thaumatin. Frame rejection was performed for each assembled dataset to different extents as described in the *Methods*: ∞, no frame rejection and 500–50%, where 50% is the most stringent rejection. (*a*) CC_1/2_ at 4 Å *d*_min_. (*b*) *R*_split_ at 4 Å *d*_min_. (*c*) Anomalous correlation coefficient (ACC) at 5 Å *d*_min_. ACC is reported as ‘DelAnom correlation between half-sets’ in the *AIMLESS* log file.

To exclude outlier crystals in the assembled data, we performed crystal rejection one by one starting from the 17-crystal dataset. This step removes the statistically least compatible crystals (Guo *et al.*, 2019 ▸). After each crystal rejection, we performed frame rejection at six ratios (Guo *et al.*, 2019 ▸). Fig. 2 ▸ shows statistics values of each dataset after crystal rejection as well as frame rejection reported by *AIMLESS*. The overall CC_1/2_ values of no-frame rejection are beyond 0.95, indicating that initial frame rejection in the single-crystal stage was effective in removing radiation-damaged frames. Stricter frame rejection, for example at a level of 50% decay (see the definition of decay in the *Methods* [Sec sec2]), showed reduced CC_1/2_ [Fig. 2 ▸(*a*)] and increased *R*_split_ values [Fig. 2 ▸(*b*)]. The anomalous signals increased significantly as the number of assembled crystals increased [Fig. 2 ▸(*c*)] up to 15, but reduced significantly for over 15 crystals, indicating that two statistical outlier crystals deteriorated the overall data quality. Taken together, our assembly and rejection strategies are highly effective for enhancing anomalous signals.

**Figure 3:**
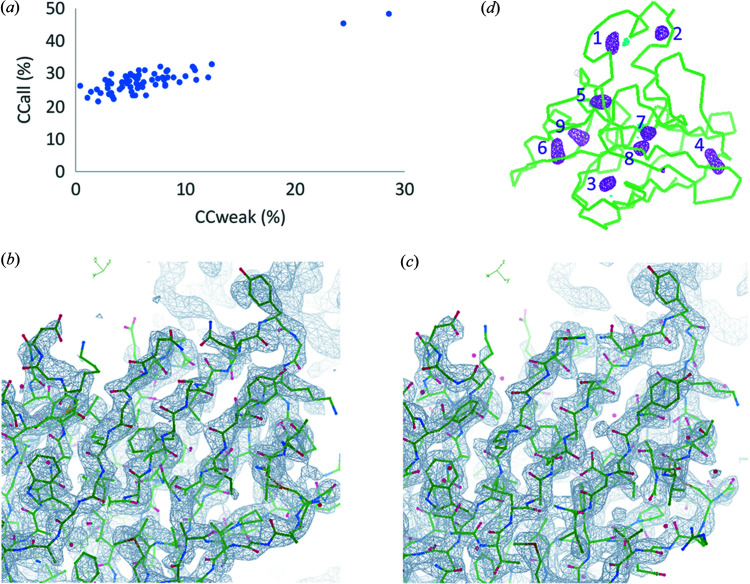
Structure determination and phasing of thaumatin. (*a*) CC_all_/CC_weak_ of *SHELXD* trials. (*b*) Experimental electron density after density modification. (*c*) Refined electron-density map. (*d*) Bijvoet-difference Fourier peaks. Peaks for anomalous scatters are shown as magenta isomeshes contoured at 4σ. The numbers indicate the positions of sulfur atoms of anomalous scatters in the structure: 1, C149–C158; 2, C159–C164; 3, C9–C204; 4, C121–C193; 5, C134–C145; 6, C56–C66; 7, C126–C177; 8, M122; 9, C71–C77. The overall structure model of thaumatin from native-SAD is shown as ribbons.

We performed native-SAD phasing for the 15-crystal dataset. With an *E*_min_ cutoff at 1.3 and resolution cutoff at 3.9 Å, we obtained a substructure solution including 9 sulfur peaks from as few as 80 *SHELXD* trials with the highest CC_all_ and CC_weak_ of 48.6 and 28.7%, respectively [Fig. 3 ▸(*a*)]. This substructure was used for SAD phasing using *PHASER*. After density modification using *PARROT*, the electron density map is of high quality at 2.6 Å resolution for model building [Fig. 3 ▸(*b*)]. *BUCCANEER* was able to build 206 out of 207 residues automatically. The refined structure has an *R*/*R*_free_ of 0.19/0.23 (Table 1 ▸), indicating high quality [Fig. 3 ▸(*c*)]. The Bijvoet-difference Fourier peaks clearly identified all sulfur peaks beyond 4.0σ [Fig. 3 ▸(*d*)]. In summary, we successfully solved the thaumatin structure at 5 keV from 15 microcrystals using the helium path.

### 3.3. Native-SAD phasing of TehA

**Figure 4:**
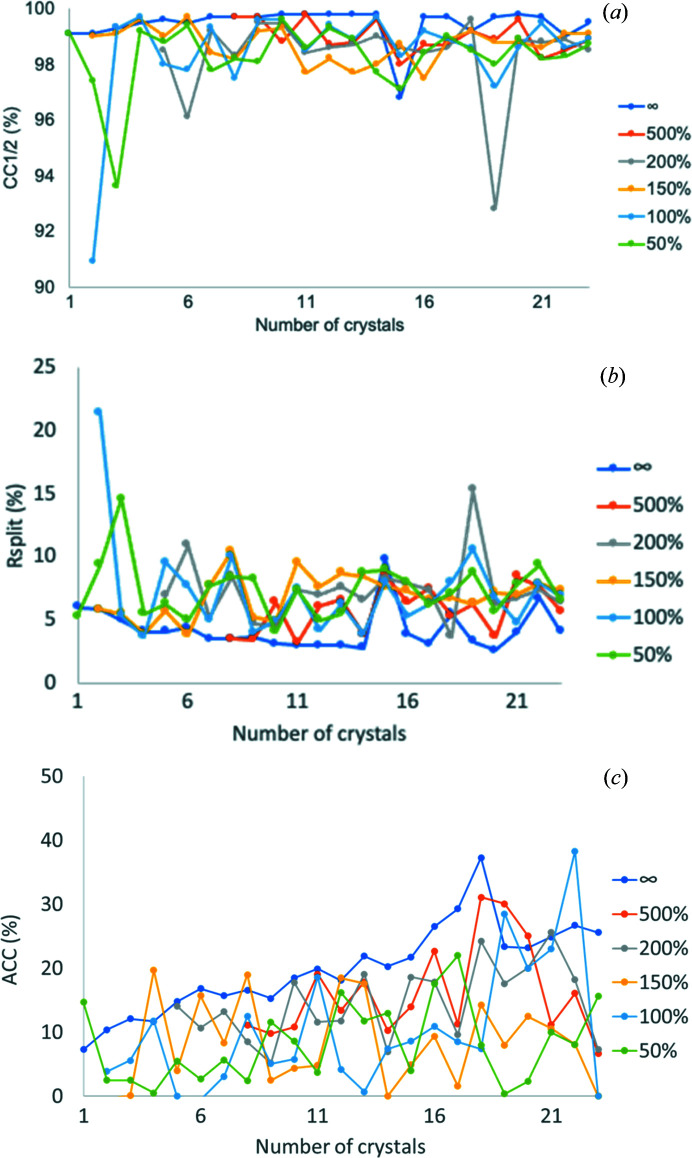
Data analysis of assembled TehA data. Frame rejection was performed for each assembled dataset to different extents as described in the *Methods*: ∞, no frame rejection and 500–50% where 50% is the most stringent rejection. (*a*) CC_1/2_ at 4 Å *d*_min_. (*b*) *R*_split_ at 4 Å *d*_min_. (*c*) ACC at 5 Å *d*_min_. ACC is reported as ‘DelAnom correlation between half-sets’ in the *AIMLESS* log file.

TehA consists of 314 residues, including 1 cysteine and 10 me­thio­nines. The calculated Bijvoet difference ratio is 1.8% at 5 keV. We applied the developed native-SAD strategy based on the approach described for thaumatin for solving a challenging case of a membrane protein TehA. We collected 23 datasets, each from a single TehA microcrystal. After rejection of radiation-damaged frames, 24 480 out of 41 400 frames were selected for data assembly in *PyMDA*. TehA crystals can be classified into two groups [Fig. S2 ▸(*b*), named uc1 and uc2], but we could not obtain enough anomalous signals for phasing from assembled data in either uc1 or uc2. We then combined uc1 and uc2 and merged 23 datasets to further enhance anomalous signals through increased multiplicity. To remove statistically incompatible crystals, we performed crystal rejection. After rejection of 5 crystals out of 23, the 18-crystal dataset showed excellent CC_1/2_ and *R*_split_ values, indicating high data quality [Figs. 4 ▸(*a*) and 4(*b*)]. The 18-crystal dataset also has the highest anomalous signals [Fig. 4 ▸(*c*)]. Because we had already rejected radiation-damaged frames during the single-crystal frame selection, the dataset from the 18 crystals was scaled and merged without the need for further frame rejection [Fig. 4 ▸(*c*)].

**Figure 5:**
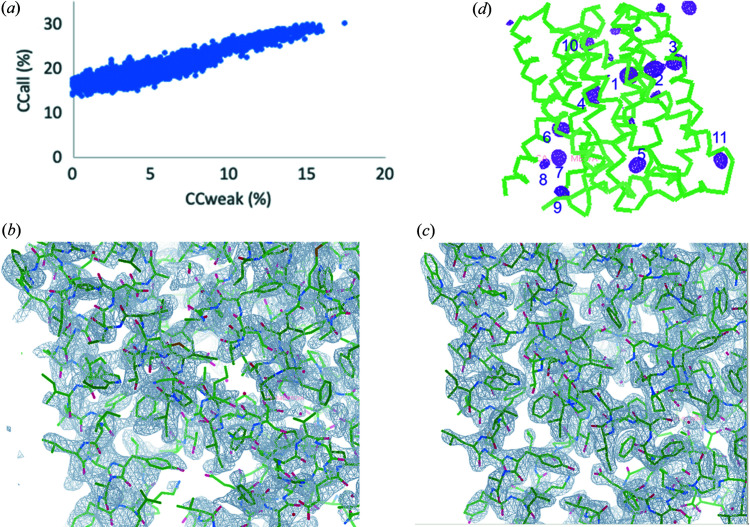
Structure determination for TehA. (*a*) CC_all_/CC_weak_ of *SHELXD* trials. (*b*) Experimental electron density after density modification. (*c*) Refined electron-density map. (*d*) Bijvoet-difference Fourier peaks. Peaks for anomalous scatters are shown as magenta isomeshes contoured at 4σ. The numbers indicate the positions of anomalous scatters in the structure: 1–9, sulfur from protein; 1, M91; 2, Cys211; 3, M174; 4, M267; 5, M201; 6, M294; 7, M58; 8, M301. Peaks 9–11 are not from protein sulfur atoms. The overall structure model of the TehA protomer from native-SAD is shown as ribbons.

Using the 18-crystal dataset, we searched for a substructure using *SHELXD*. We obtained promising solutions from extensive optimization of *SHELXD* parameters of high-resolution cutoff, minimum normalized *E* values (*E*_min_) and number of substructures. The *SHELXD* parameters used for finding a solution are a high-resolution cutoff of 3.2, *E*_min_ = 1.7 and 5 anomalous scatterers. We obtained a substructure solution with the highest CC_all_ and CC_weak_ of 35.4 and 25.8%, respectively [Fig. 5 ▸(*a*)]. This substructure was used for SAD phasing using *PHASER*. After density modification by *PARROT*, we obtained an electron-density map with sufficient quality for model building [Fig. 5 ▸(*b*)]. *BUCCANEER* was able to build 300 out of 314 residues automatically. The refined structure has an *R*/*R*_free_ of 0.17/0.22, indicating high map quality [Fig. 5 ▸(*c*)]. In the Bijvoet-difference Fourier map, we found 8 sulfur peaks [Fig. 5 ▸(*d*), peaks 1–8] as well as unknown peaks [Fig. 5 ▸(*d*), peaks 9–11]. With TehA as an example, we suggest that low-energy native-SAD phasing using a helium path could be used more routinely for solving challenging membrane protein structures.

## 4. Discussion

### 4.1. Radiation damage

**Figure 6:**
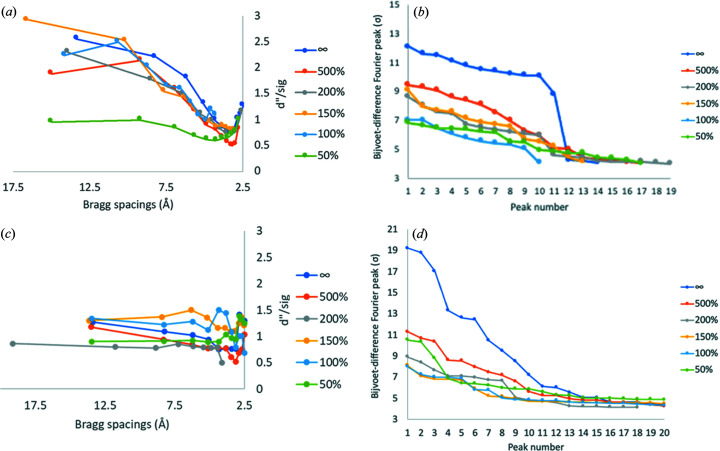
Analysis of assembled 15-crystal thaumatin data and 18-crystal TehA data. (*a*) Plot of *d*″/sig with respect to *d*_min_. *SHELXC* was used for calculation of the anomalous signal from 15 assembled thaumatin crystals with and without frame rejection. (*b*) Plot of the 19 highest Bijvoet-difference Fourier peaks of thaumatin. Total frames, 18900; rejected frame numbers: 500%, 425; 200%, 7388; 150%, 9152; 100%, 11370; 50%, 14796. (*c*) Plot of *d*″/sig with respect to *d*_min_. *SHELXC* was used for the calculation of anomalous signals from 18 assembled TehA crystals with and without frame rejection. (*d*) Plot of the 20 highest Bijvoet-difference Fourier peaks of TehA. Total frames, 24480; rejected frame numbers: 500%, 285; 200%, 2538; 150%, 4897; 100%, 9424; 50%, 15338.

In this work we used a helium path to increase signal relative to noise for low-energy X-ray diffraction. Due to radiation damage, we rejected about half of the frames collected to enhance anomalous signals for native-SAD phasing. A previous study showed that the frame-rejection step in *PyMDA* further enhanced the anomalous signals (Guo *et al.*, 2019 ▸), but we did not observe such enhancement here (Figs. 2 ▸ and 4 ▸). To investigate this, we examined the impact of frame rejection at different extents on the best assembled datasets for thaumatin [Figs. 6 ▸(*a*) and 6(*b*)] and TehA [Figs. 6 ▸(*c*) and 6(*d*)]. In both cases, we found that anomalous signals as well as individual Bijvoet difference peak heights were dramatically reduced as the extent of frame rejection (*e.g.* decay parameter) increased. Since radiation-damaged frames were already rejected in our initial single-crystal frame rejection step, as expected the subsequent frame rejection in *PyMDA* did not improve the assembled data quality.

### 4.2. Multi-crystal data assembly and anomalous signals

Both the thaumatin and TehA structures were determined using native-SAD with data combined from microcrystals. We collected complete datasets for each crystal. As shown in Tables S1 and S2 of the supporting information, each single-crystal dataset is more than 90% complete with a multiplicity ranging from 2.4 to 22.7 for thaumatin and 3 to 10.3 for TehA. However, we did not observe apparent anomalous signals at the single-crystal level. This is probably not surprising as even at a low-energy of 5 keV with a helium environment, diffraction data from a 20 µm crystal were not sufficient for native-SAD phasing. To enhance *f*″ of sulfur, Aurelius *et al.* (2017 ▸) used a near sulfur *K*-edge at 2.5 keV for native-SAD phasing of thaumatin. However, the large Bragg angles at this energy only allowed collection of diffraction data at 3.2 Å resolution using a flat detector (Aurelius *et al.*, 2017 ▸), and a large, curved detector is required to collect such high-angle diffraction spots (Wagner *et al.*, 2016 ▸). Here, we took our established strategy to enhance sulfur *f*″ by multi-crystal assembly and rejection (Liu *et al.*, 2012 ▸). For this method, it is essential to select compatible crystals for assembly. Our unit-cell variation analysis revealed that all crystals of thaumatin are compatible with each other [Fig. S2 ▸(*a*)]. As a comparison, TehA crystals can be classified into two groups [Fig. S2 ▸(*b*)]. The biggest differences are no more than 2 Å in each dimension (Table S2). Data assembled from each group were not sufficient for native-SAD phasing. For native-SAD phasing, the low-resolution anomalous signals are more important and are less affected by crystal non-isomorphism, so we combined the two groups. We could then obtain high-quality data after rejection of five outlier crystals, suggesting that our crystal-rejection step can effectively remove incompatible crystals spreading in two groups [Fig. S2(*b*)]. The anomalous signals increased as the number of compatible crystals increased, and a total of 18 TehA crystals were sufficient for native-SAD phasing.

We also evaluated the strength of anomalous signals after *f*″ refinement (Liu *et al.*, 2013 ▸). For thaumatin, the highest *f*″ value was 1.40 e for Cys9 and Cys204, and the lowest *f*″ was 0.91 e for Cys126 and Cys177, with an average of 1.23 e. The average value was close to the theoretical *f*″ value 1.31 for sulfur, indicating that the majority of the anomalous signals (94%) were preserved at 5 keV with a helium path. For TehA, the highest *f*″ value of sulfur was 1.66 e for Met174, and the lowest *f*″ was 0.80 e for Met201, with an average of 1.05 e. We did not find Bijvoet-difference Fourier peaks for Met1 and Met39. The two residues are on the protein surface and their side chains are poorly ordered.

### 4.3. Anomalous scatterers

**Figure 7:**
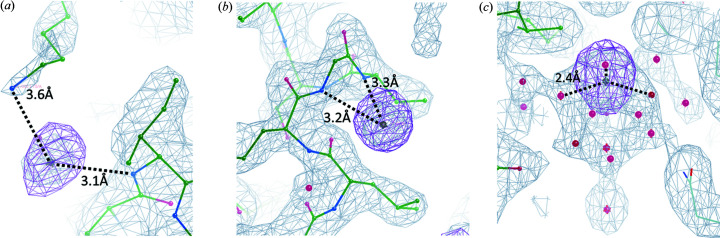
Electron densities for three non-sulfur anomalous scatterers in TehA. 2*F*_o_ − *F*_c_ electron densities are shown as gray isomeshes at 1.5σ; and Bijvoet-difference Fourier peaks are shown as purple isomeshes at 4.0σ. The refined TehA model is shown as sticks. (*a*) Peak 9. (*b*) Peak 10. (*c*) Peak 11.

In low-energy native-SAD phasing, non-sulfur/phospho­rus anomalous scatterers may be found at a position previously identified as water or a light element (Basu, Finke *et al.*, 2019 ▸). These unknown anomalous scatterers can provide extra information that is not available in conventional crystallographic structure refinement. In this work, we found three unknown Bijvoet-difference Fourier peaks in TehA that were not sulfur [Fig. 6 ▸(*d*), peaks 9–11]. Our crystallization conditions (reservoir and protein solution) include Na^+^, Cl^−^, Zn^2+^, P and SO_4_^2−^. We assume that these unknown peaks may come from the anomalous scatterers. To identify the three anomalous scatterers, we performed *f*″ refinement using *phenix.refine* (Liu *et al.*, 2013 ▸). We found approximate *f*″ values were 1.66 for peak 9, 1.67 for peak 10 and 0.63 for peak 11. The *f*″ values of peaks 9 and 10 were very close to the calculated *f*″ values of Cl^−^ and Zn^2+^ at 5 keV. The anomalous peaks 9 and 10 are coordinated by a nearby nitro­gen atom of the main chain or a Lys residue within a 3.2 Å distance [Figs. 7 ▸(*a*) and 7(*b*)], revealing that the two peaks should come from Cl^−^ but not Zn^2+^. Peak 11 is not coordinated by any protein atom but seems to be hydrated by water molecules [Fig. 7 ▸(*c*)] and has an *f*″ value close to the calculated value of Na^+^ at 5 keV, suggesting that peak 11 could be Na^+^.

TehA is an uncharacterized candidate for an ion channel as it is a bacterial homolog of the plant nitrate/chloride channel SLAC1 (Deng *et al.*, 2021 ▸). We did not observe any of the three anomalous peaks in previous structures using higher energy X-rays (Chen *et al.*, 2010 ▸). Our present work used low-energy X-ray scattering at 5 keV which produced much higher anomalous scattering signals for Cl^−^ and Na^+^, leading to the identification of both ions bound to TehA. Interestingly, Na^+^ is located on the threefold axis of the TehA trimer, suggesting that Na^+^ may possibly be involved in TehA function. Our analyses thus provide additional information on the identification of potential ions that might modulate the function of TehA and its homologs.

## 5. Concluding remarks

Native-SAD phasing using <6 keV X-rays is challenging because of substantial background scattering and absorption issues. Here we used a helium path to enhance weak anomalous signals at 5 keV from microcrystals at the NSLS-II FMX beamline. We demonstrated that *de novo* structure determination of a membrane protein by native-SAD phasing from as few as 18 micrometre-sized crystals is feasible using a helium-path environment at 5 keV. The combination of helium-path data collection with a multi-crystal data processing strategy including iterative assembly and rejection, dramatically reduces the number of crystals required, and thereby greatly increases the viability of native-SAD phasing for solving challenging membrane protein structures. With TehA as an example, we suggest that low-energy native-SAD phasing using a helium path could be used more routinely for solving challenging membrane protein structures.

Atomic coordinates and structure factor files have been deposited in the RCSB Protein Data Bank (PDB) under the accession codes 8ena for thaumatin and 8en9 for TehA.

## Supplementary Material

Supporting tables and figures. DOI: 10.1107/S205225252200971X/lz5060sup1.pdf

PDB reference: 8ena

PDB reference: 8en9

## Figures and Tables

**Table 1:** Data collection and refinement statistics

	Thaumatin	TehA
Data collection		
Beamline	FMX (NSLS-II)	FMX (NSLS-II)
Wavelength (Å)	2.48	2.48
Space group	*P*4_1_2_1_2	*R*3
*a*, *c* (Å)	57.6, 150.2	95.4, 136.1
Solvent content (%)	56.3	63.6
Bragg spacings (Å)	39.3–2.5 (2.53–2.5)	39.5–2.6 (2.7–2.6)
Total reflections	1696197 (557)	1550748 (43309)
Bijvoet unique reflections†	16225 (372)	14153 (1719)
Completeness (%)	100.0 (100.0)	99.8 (98.3)
〈*I*/σ(*I*)〉	20.0 (0.9)	24.4 (3.5)
*R*_split_	0.061 (1.79)	0.041 (0.48)
Multiplicity	183.6 (3.0)	109.6 (25.2)
CC_1/2_	0.979 (0.065)	0.995 (0.482)
Refinement		
Resolution (Å)	2.6	2.6
No. of reflections	16153 (1109)	14132 (1352)
*R*_work_/*R*_free_	0.185/0.234	0.170/0.217
No. of atoms	1668	2580
Wilson *B* factor (Å^2^)	24.7	35.19
Average *B* factor (Å^2^)	25	34
R.m.s. deviations		
Bond length (Å)	0.005	0.008
Bond angle (°)	0.795	0.83
PDB code	8ena	8en9

† The numbers of unique reflections with Bijvoet pairs separated are given in parentheses. Elsewhere in the table, values in parentheses are for the highest resolution range.
