# Supplementary material for: Multi-crystal native-SAD phasing at 5 keV with a helium environment
Source: IUCrJ. 2022 Oct 21;9(Pt 6):768–77. doi: 10.1107/S205225252200971X (PMC9634608; doi:10.1107/S205225252200971X)
Supplement: Supplementary file 1 [file m-09-00768-sup1.pdf]

IUCrJ

Volume 10 (2023)

Supporting information for article:

Multi-crystal native-SAD phasing at 5 keV with a helium environment

Akira Karasawa, Babak Andi, Martin R. Fuchs, Wuxian Shi, Sean McSweeney, Wayne A. Hendrickson and Qun Liu

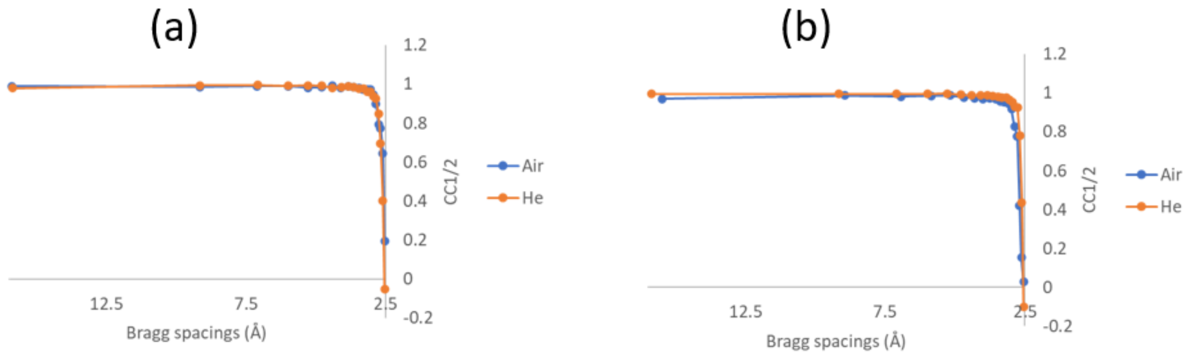

Supplemental Figure 1. Comparison of best single crystal data set collected under helium and air. (a) Plot of $CC_{1/2}$ against d_{min} for thaumatin. (b) Plot of $CC_{1/2}$ against d_{min} for TehA.

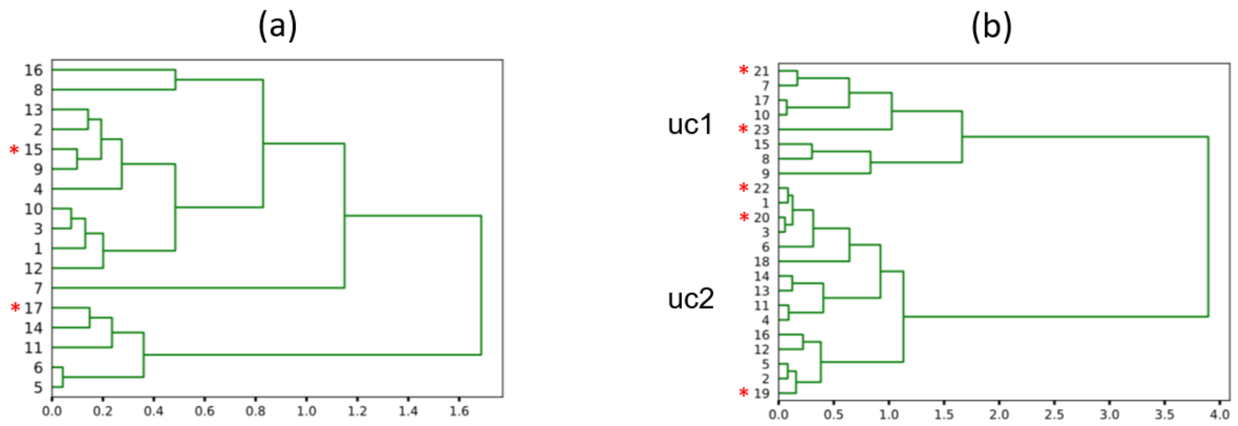

Supplemental Figure 2. Unit-cell variation analysis for classification of single-crystal data set of thaumatin (a) and TehA (b). Crystals rejected from the final data are indicated by a red asterisk.

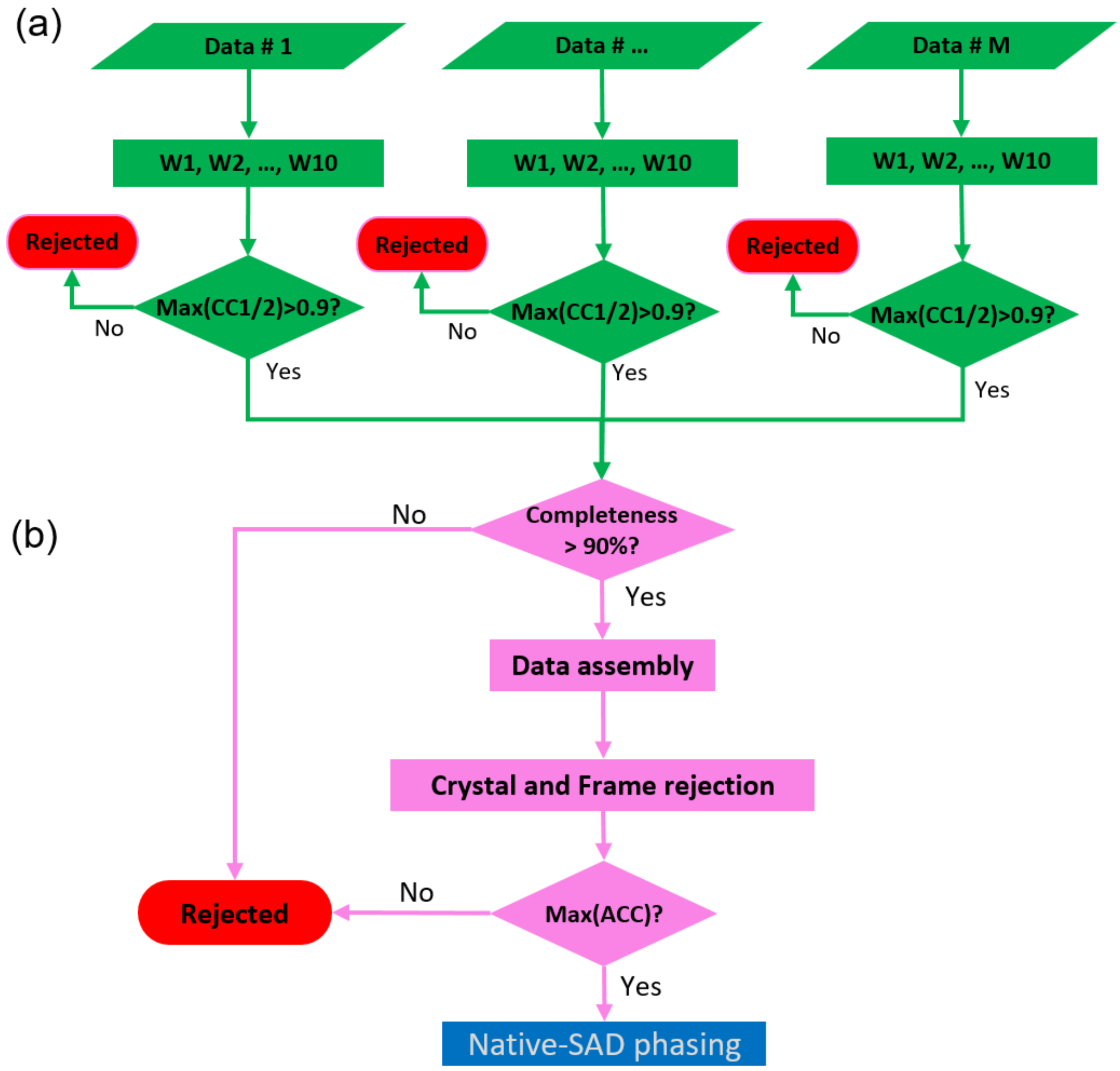

Supplemental Figure 3. Schematic diagram of data processing. (a) Single-crystal data processing. M single crystal data set (19 for Thaumatin, and 26 for TehA) were independently indexed, integrated and scaled using PyMDA as 10 progressive wedges (W1, 1-180; W2, 1-360; …, W10, 1-1800 frames). Only best wedge per data set which showed $CC_{1/2}$ >0.9 were selected for next step (17 for Thaumatin, and 23 for TheA were selected). (b) Crystal assembly and rejection. Combined data sets were subjected to crystal and frame rejection by PyMDA. Two crystals were rejected for thaumatin and 5 crystals were rejected for TehA. Assembled data with the highest ACC were selected for native-SAD phasing.

Supplemental Table 1. Statistics of individual data sets collected for thaumatin.
Crystal numbers are corresponding to the dendrograms in supplemental figure 2.

Crystal number	Frame selected	Unit cell dimensions a, c (Å)	Completeness (%)	<I/σ(I)>	R_{split}	Multiplicity	$CC_{1/2}$
1	1-1620	57.79, 150.27	99.9	16.3	0.058	20.6	0.993
2	1-720	57.66, 150.17	99.9	17.3	0.054	9.1	0.994
3	1-1800	57.69, 150.24	99.8	19.7	0.050	22.7	0.993
4	1-540	57.77, 150.03	99.9	17.0	0.061	6.8	0.989
5	1-1620	57.50, 149.86	99.9	9.7	0.103	20.5	0.955
6	1-360	57.49, 149.82	98.3	14.1	0.071	4.6	0.989
7	1-1800	57.89, 151.01	99.9	14.7	0.065	22.7	0.988
8	1-180	57.31, 150.36	94.2	12.3	0.074	2.4	0.985
9	1-1260	57.61, 150.01	99.8	10.2	0.086	15.9	0.985
10	1-1800	57.73, 150.29	99.9	12.9	0.076	22.8	0.983
11	1-1800	57.29, 149.85	99.9	11.3	0.098	22.7	0.959
12	1-1800	57.66, 150.39	99.9	10.7	0.089	22.8	0.982
13	1-1620	57.56, 150.16	99.8	12.0	0.084	20.5	0.980
14	1-360	57.41, 149.74	93.0	10.0	0.090	5.0	0.976
15	1-1620	57.67, 150.06	99.9	8.7	0.103	20.5	0.973
16	1-900	57.59, 150.64	99.9	5.5	0.151	11.3	0.957
17	1-1440	57.34, 149.63	99.9	6.3	0.171	18.2	0.918

Supplemental Table 2. Statistics of individual data sets collected for TehA. Crystal numbers are corresponding to the dendrograms in supplemental figure 2.

Crystal number	Frame selected	Cell dimensions a, a, c (Å)	Completeness (%)	<I/σ(I)>	R_{split}	Multiplicity	$CC_{1/2}$
1	1-900	95.24, 135.87	99.8	24.4	0.036	5.1	0.997
2	1-1800	94.92, 136.01	99.8	15.2	0.057	10.3	0.995
3	1-900	95.28, 135.85	99.9	19.0	0.047	5.1	0.995
4	1-720	95.07, 136.20	99.8	17.1	0.048	4.1	0.994
5	1-1800	94.88, 135.95	99.8	16.6	0.054	10.2	0.993
6	1-1080	95.45, 135.87	99.9	12.2	0.071	6.1	0.992
7	1-1800	95.82, 136.23	99.9	14.2	0.067	10.3	0.992
8	1-1620	96.03, 136.68	99.7	8.1	0.091	9.2	0.991
9	1-1800	96.54, 136.54	99.9	10.8	0.099	10.2	0.99
10	1-1260	95.58, 136.47	99.8	12.7	0.074	7.2	0.989
11	1-1620	95.07, 136.11	99.9	13.5	0.068	9.2	0.989
12	1-900	94.95, 135.64	99.9	8.0	0.103	5.1	0.987
13	1-1620	95.28, 136.26	99.7	7.7	0.106	9.2	0.987
14	1-1620	95.26, 136.14	99.8	9.5	0.095	9.2	0.983
15	1-1620	96.11, 136.96	99.9	7.2	0.128	10.2	0.982
16	1-1620	95.05, 135.81	99.7	7.4	0.112	9.2	0.983
17	1-1800	95.63, 136.45	99.9	10.2	0.094	10.2	0.985
18	1-1620	95.25, 135.39	99.6	8.4	0.103	9.2	0.980
19	1-1080	94.93, 135.85	99.9	6.6	0.133	6.1	0.977
20	1-1620	95.32, 135.86	99.7	8.6	0.11	9.2	0.972
21	1-1620	95.94, 136.23	99.9	6.2	0.225	9.3	0.949
22	1-540	95.26, 135.95	99.8	6.5	0.181	3.0	0.94
23	1-720	95.81, 135.54	99.8	5.7	0.212	4.1	0.903
